# Notes

**Published:** 1896-11

**Authors:** 


### Notes.

### THE COLLEGE OPENINGS.

The Southern Medical College opened Thursday, October 1, with a gratifyingly large attendance. The college building has undergone a number of important improvements. A marble floor has replaced the old wooden one of the amphitheatre, making it one of the finest lecture-rooms in any institution in the South.

The changes in the faculty have resulted in making an uncommonly strong combination, and everything points to a successful year.

The opening exercises of the Atlanta Medical College took place October 7. The number of new students enrolled was more than usually large. The most important improvement in the college consists in the addition of the new and magnificently equipped pathological laboratory. It occupies a separate building and is equipped with all the necessary apparatus for carrying on pathological and bacteriological work. Dr. Blackford, the instructor, expects to make the course attractive and will from time to time give magic lantern exhibitions of photo-micrographs illustrating the salient points in pathology and bacteriology.

### ANTITOXIN COLLECTIVE INVESTIGATION.—AMERICAN PEDIATRIC SOCIETY.

We are requested to publish the following:

*To the Profession:*

The American Pediatric Society are encouraged to ask the co-operation of the profession in a further collective investigation. Laryngeal diphtheria is believed to furnish a crucial test for antitoxin; the present aim is to ascertain (1) what percentage of cases of laryngeal diphtheria recover without operation, under antitoxin treatment; (2) what percentage of operated cases recover.

The Society asks for records of cases of *diphtheria involving the larynx, whether operated or not, occurring in private practice in the United States and Canada, treated with antitoxin.* It is expected that cases occurring this year will be treated with reliable preparations of the serum, will be treated early, and will be given efficient doses. The second report is designed to be a study of cases occurring between the closing of the first report, May 1st, 1896, and the closing of the present collective investigation, April 1st, 1897.

In order to secure data which shall make the tables complete, circulars containing blanks for ten cases have been printed and are now ready for distribution. It is desired that physicians shall fill out circular, blanks, as cases occur, not trusting to memory, and shall urge their friends having similar cases to do the same. Circulars can be had by applying to the committee (address below). Several groups of cases in the first investigation arrived too late and were lost to the report. It is desired that circulars as soon as filled, (ten cases,) be returned to the committee. The collection of cases must close at the end of March, 1897.

For extra circulars (blanks) for returning circulars (filled), and for further information, address the chairman of the committee, W. P. Northrup, M. D., No. 57 East 79th Street, New York, N. Y.

---

(1) *Dosage.* For a child over two years old, the dosage of antitoxin should be in all laryngeal cases with stenosis, and in all other severe cases, 1500 to 2000 units for the first injection, to be repeated in from eighteen to twenty-four hours if there is no improvement; a third dose, after a similar interval, if necessary. For severe cases in children under two years, and for mild cases over that age, the initial dose should be 1000 units, to be repeated as above, if necessary; a second dose is not usually required. The dosage should always be estimated in antitoxin units and not of the amount of serum.

(2) *Quality of antitoxin.* The most concentrated strength of an absolutely reliable preparation.

(3) *Time of administration.* Antitoxin should be administered as early as possible on a clinical diagnosis, not waiting for a bacteriological culture. However late the first observation is made, an injection should be given, unless the progress of the case is favorable and satisfactory.

---

### ATLANTA MEN COMPLIMENTED.

At the recent meeting of the Tri-State Medical Society at Chattanooga, Dr. W. F. Westmoreland was elected president of the Association, and Dr. Miller B. Hutchins vice-president for Georgia.

---

RESIGNED FROM THE ARMY.—DR. HAMILTON REFUSES TO LEAVE CHICAGO FOR SAN FRANCISCO.—Dr. John B. Hamilton, Surgeon-General of the United States Marine Hospital, stationed in this city, has sent in his resignation to President Cleveland. This action is the outcome of a long and bitter fight in the higher circles of the War Department.

Some time ago Dr. Hamilton was ordered to the Marine Hospital at San Francisco by the War Department.

His headquarters have been in Chicago since 1891, and he has a large and valuable private practice, as well as real estate holdings. Owing to these circumstances, he objected to being transferred, and made an official protest. This protest was overruled and Dr. Hamilton was again ordered West. He then protested to Secretary Carlisle, and after waiting until Sept. 24, was notified that the Secretary would not interfere. He then decided to resign, and on Monday took that action. He has not yet received a reply.

Dr. Hamilton blames Surgeon-General Walter Wyman for the objectionable order.—*New York Times.*

---

DR. C. E. BROWN-SEQUARD, a son of the noted French scientist, died recently in Atlanta, where he had resided for the last three years. It will be remembered that Brown-Sequard was an American.

### Book Reviews

PRACTICAL DIAGNOSIS. The Use of Symptoms in the Diagnosis of Disease. By Hobart Amory Hare, M. D., B. Sc., Professor of Therapeutics in Jefferson Medical College of Philadelphia; Physician to the Jefferson Medical College Hospital; Laureate of the Medical Society of London, of the Royal Academy in Belgium; Corresponding Fellow of the Sociedad Espanol de la Higiene of Madrid; Member of the Association of American Physicians, etc. Illustrated with 191 engravings and 13 colored plates. Philadelphia and New York: Lea Brothers & Co. 1896.

This work is a departure from the usual methods of treating the subject of diagnosis. Prominent symptoms are seized upon and applied to the disease with which they are connected, and a diagnosis thus inductively established. The utility of this plan can be at once perceived.

The marked symptoms referable to the different organs are systematically set forth. The volume is doubly indexed, having one index of symptoms, another of diseases. The author's reputation for accurate observation and medical acumen is well sustained in this latest work.

TREATMENT OF APPENDICITIS. By John B. Deaver, M. D., Surgeon to the German Hospital, Philadelphia. Containing 32 full-page plates and other illustrations. Philadelphia. P. Blakiston, Son & Co. 1896. Cloth 8vo. Pages 168. Price $3.50.

This volume is a systematic discussion of this much discussed subject, based on an experience of five hundred cases. The author takes the ground that appendicitis should be operated upon when the diagnosis is conclusive, but if the operation can not be performed for any reason, he counsels the use of conservative measures, or laxatives and turpentine stupes. The chapter on differential diagnosis is very full, and includes all the phases of atypical cases. The operative steps are thoroughly illustrated and minutely described. The book will doubtless prove of value to the surgeon, but is somewhat too radical for the general practitioner.

THE THERAPEUTIC APPLICATIONS OF THE PEROXIDE OF HYDROGEN (MEDICINAL) GLYCOZONE, HYDROZONE AND EYE BALM. By Charles Marchand, Chemist, graduate of the "Ecole Centrale Des Arts et Manufactures De Paris," France. Eleventh edition. New York.

This volume of 216 pages represents the collective information on the therapeutic uses of these very valuable remedies.

The number of diseased conditions successfully treated with peroxide of hydrogen grows larger as our knowledge of the drug increases. There is much in the book that will be of interest to the professional reader. The volume can be obtained by writing to Charles Marchand, Chemist, 28 Prince Street, New York.

---

QUIZ COMPENDS No. 7. A Compend of Gynecology. By William H. Wells, M. D., Adjunct Professor of Obstetrics and Diseases of Infancy in the Philadelphia Polyclinic; late Assistant Demonstrator of Clinical Obstetrics in the Jefferson Medical College, Philadelphia, etc. With 150 illustrations. Philadelphia: P. Blakiston, Son & Co., 1012 Walnut St. 1896. For sale by James F. Meegan, Atlanta.

This little work is one of the latest of this very useful series of compends. It will be found a valuable aid to the student as well as to the practitioner who wishes to refresh himself with the subject. It is aptly illustrated throughout.

---

THE STUDENT'S MEDICAL DICTIONARY. Including all the words and phrases generally used in medicine, with their proper pronunciation and definitions based on recent medical literature. By George M. Gould, A. M., M. D., author of "An Illustrated Dictionary of Medicine," "Biology, and Allied Sciences," "12,000 Medical Words Pronounced and Defined," "The Meaning and the Method of Life," "Borderland Studies;" formerly editor of the *Medical News;* President, 1893-1894, American Academy of Medicine. With elaborate tables of the Bacilli, Micrococci, Leucomaines, Etc., of the Arteries, Ganglia, Muscles, and Nerves; of Weights and Measures, Analyses of the Waters of the Mineral Springs of the United States, etc., etc. Tenth edition, re-written and enlarged. Philadelphia: P. Blakiston, Son & Co., 1012 Walnut Street. 1896. Price, $3.25. For sale by James F. Meegan, Atlanta, Ga.

Though called the tenth edition of the Standard Medical Dictionary, this volume, according to the author, is entirely new. It is of handy size for ready reference, and contains many tables of nerves, muscles, etc., with an appendix devoted to the analyses of the mineral springs of this country. A unique feature of this dictionary is a comprehensive table of eponymic diseases—that is, those diseases which have received the name of the writer who was first to describe them.

The binding and presswork of the book are excellent.

